# Use of Chemoimmunotherapy for Locally Advanced Deficient Mismatch Repair (dMMR) Gastric Adenocarcinoma With Curative Intent: A Case Report

**DOI:** 10.7759/cureus.63527

**Published:** 2024-06-30

**Authors:** Beatriz Gonzalez Diez, Maria Jesus Fernandez Aceñero, Ramiro Jesus Mendez, Esteban Martin-Antona, Javier Sastre

**Affiliations:** 1 Medical Oncology, Hospital Clínico San Carlos, Madrid, ESP; 2 Pathology, Hospital Clínico San Carlos, Madrid, ESP; 3 Radiodiagnosis, Hospital Clínico San Carlos, Madrid, ESP; 4 General Surgery, Hospital Clínico San Carlos, Madrid, ESP; 5 Oncology, Hospital Clínico San Carlos, Madrid, ESP

**Keywords:** dmmr, msi-h, microsatellite instability, immunotherapy, gastric cancer

## Abstract

The standard of care for patients with operable gastric adenocarcinoma is perioperative chemotherapy and surgical resection. The deficient mismatch repair (dMMR)/microsatellite instability (MSI-H) phenotype is a major predictive biomarker for immune checkpoint inhibitors (ICIs) efficacy in advanced disease. Several phase II and III trials suggest a promising future role of immunotherapy with or without chemotherapy in the neoadjuvant/adjuvant setting, especially in MSI-H localized gastric adenocarcinomas. We present a 38-year-old man diagnosed in March 2022 with poorly differentiated gastric adenocarcinoma clinical stage III (cT4 N0 M0) with deficiency of MLH1 and PMS2, combined positive score (CPS) of 100 and negative HER2 immunohistochemistry, had poor tumor response to preoperative 5-FU, leucovorin, oxaliplatin, and docetaxel (FLOT). It was considered unresectable because of the involvement of the colon, mesocolon, duodenum, pancreas, and retroperitoneum. Then, first-line systemic treatment with 5-FU, leucovorin, and oxaliplatin (FOLFOX)-nivolumab was initiated in August 2022, with a significant radiologic tumor reduction after six cycles, allowing a curative surgery in March 2023 with complete pathologic tumor response, followed by capecitabine and nivolumab for one year, maintaining radiological remission in the last follow-up in April 2024. With this case report, we conclude that it is likely that chemoimmunotherapy or immunotherapy alone may be alternative neoadjuvant treatment choices for MSI-H locally advanced gastric cancer patients.

## Introduction

Gastric cancer is the fifth leading cause of cancer worldwide, with 75% of cases diagnosed in Asia. The standard of care for patients with resectable gastric adenocarcinoma is perioperative chemotherapy and surgical resection, based on the significant improvement in five-year survival from 23% to 36% demonstrated in the phase III MAGIC trial that compared perioperative epirubicin, cisplatin, and 5-FU (ECF) to surgery alone [1]. A similar but smaller French phase III trial evaluated perioperative cisplatin plus 5-FU, suggesting that anthracyclines may not be needed for optimal results [2]. A German phase II-III study investigating eight cycles of peri-operative 5-FU, leucovorin, oxaliplatin, and docetaxel (FLOT) versus six cycles of ECF/epirubicin, cisplatin, and capecitabine (ECX) reported a significant improvement in the primary endpoint of median overall survival (mOS) (50 versus 35 months) [3]. Based on these data, the perioperative use of FLOT should be regarded as standard of care [4].

The incidence of deficient mismatch repair (dMMR)/microsatellite instability (MSI-H) in gastric adenocarcinomas is around 22% in Western countries, and it constitutes a relevant subgroup associated with older age, female sex, distal stomach location, lower number of lymph-node metastases, and a favorable prognosis [5]. The dMMR/MSI-H phenotype has now become a major predictive biomarker for the efficacy of immune checkpoint inhibitors (ICIs) in advanced disease, including gastric and esophagogastric junction (GEJ) adenocarcinoma, independently of the tumor type [6].

These data led to the discussion on immunotherapy value in the neoadjuvant and/or adjuvant settings in routine clinical practice for patients diagnosed with locally advanced dMMR/MSI-H gastric/GEJ adenocarcinoma.

## Case presentation

A 38-year-old African-American male with eradicated *Helicobacter pylori* infection presented with epigastralgia and vomiting in March 2022, accompanied by weight loss in the last six months. A gastroscopy showed an ulcerated 7-8 cm lesion at the greater gastric curvature, suggesting a primary gastric neoplasia. Pathological studies showed a poorly differentiated gastric adenocarcinoma with a loss of nuclear expression of MLH1 and PMS2, a combined positive score (CPS) of 100, and immunochemistry for HER2 negative.

A body computed tomography (CT) scan showed the neoplastic mass in the greater gastric curvature with questionable involvement of the splenic angle of the colon and the pancreas and moderate ascites in the pelvis without evidence of distant tumor disease. The case was discussed by our Gastrointestinal Multidisciplinary Team, deciding to perform an exploratory laparoscopy, which ruled out peritoneal involvement. With the diagnosis of the poorly differentiated gastric adenocarcinoma clinical stage III (cT4 N0 M0), we initiated perioperative treatment with FLOT (docetaxel 50 mg/m^2^ + oxaliplatin 85 mg/m^2^ + leucovorin 200 mg/m^2^ + fluorouracil 2600 mg/m^2^ in continuous 24-hour infusion, every 14 days). After four cycles of FLOT, no relevant changes in the CT scan were observed.

On July 29, 2022, using a laparoscopic approach, the large gastric tumor was observed, still infiltrating the left half of the transverse colon up to the splenic angle, the pancreas, and the mesocolon. It was converted to laparotomy to complete the assessment and detect infiltration of the body and tail of the pancreas and infiltration of the angle of Treitz, distal duodenum, and retroperitoneum. Due to these findings, the lesion was considered unresectable.

**Figure 1:**
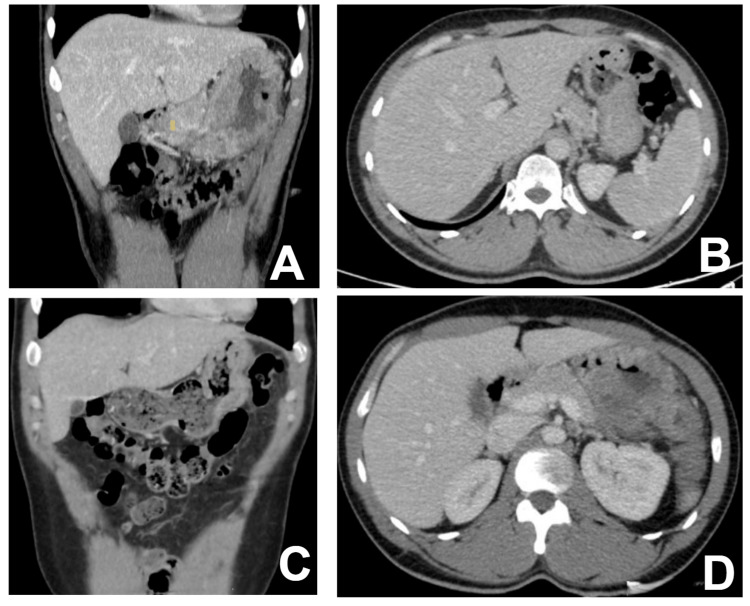
Radiological findings (A,B) First CT examination in March 2022. (C,D) Follow-up CT examination after medical treatment in January 2023. Axial (A) and coronal (B) images were acquired in a portal phase after IV iodinated contrast administration. There is a large gastric tumor presenting as an irregular mural thickening, affecting both the anterior and posterior walls of the gastric body. The inner surface of the mass is irregular due to tumor ulceration, and the posterior gastric wall shows signs of retroperitoneal infiltration with no fat plane with the pancreatic tail. No focal liver lesions or clearly enlarged lymph nodes were detected. Axial (C) and coronal (D) images were acquired in a portal phase after IV contrast injection. The gastric wall is now only minimally thickened in the upper part of the gastric body. The inner surface of the gastric wall is smooth with no clear ulcers, and there is a fat plane between the gastric wall and the pancreas with no radiological signs of retroperitoneal infiltration.

Based on the results of the CheckMate 649 study [7] for the MSI-H gastric cancer subgroup population, first-line systemic treatment with FOLFOX-nivolumab (leucovorin 400 mg/m^2^, fluorouracil 400 mg/m^2^, + 1200 mg/m^2^ in 48 hours, oxaliplatin 85 mg/m*2*, and nivolumab 240 mg every two weeks) was initiated in August 2022. After six cycles, due to an allergic reaction to oxaliplatin and leucovorin, a maintenance regimen with capecitabine and nivolumab was decided. The next CT scan assessment in January 2023 showed a significant size reduction of the gastric tumor without pancreatic infiltration (Figure 1). A re-evaluation by our Gastrointestinal Tumor Multidisciplinary Team recommended a new surgical exploration with the aim of a radical oncologic surgery attempt again.

**Figure 2:**
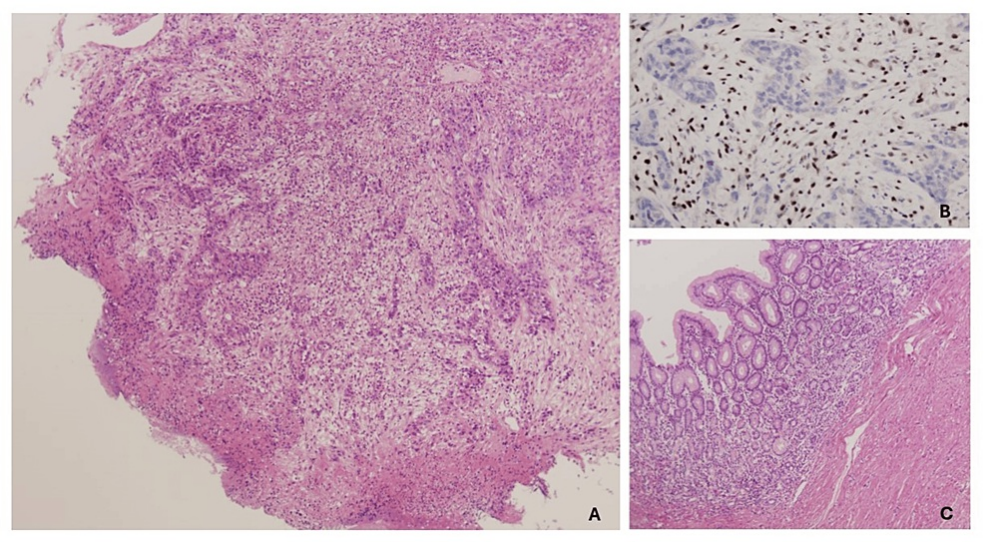
Pathological findings (A) Medium-power image of the endoscopic biopsy in March 2022, showing a neoplastic population with a trabecular and solid growth pattern (hematoxylin-eosin, ×200). (B) High-power view of the immunohistochemistry for MLH1 in the endoscopic biopsy in March 2022. Note the absence of expression in the tumor glands, which is in clear contrast with the intense staining of the cells in the lamina propria (internal control). PMS2 was also negative, and MSH2 and MSH6 were preserved (immunohistochemistry for MLH1 [Agilent, Denmark], ×400). (C) Medium-power image of the gastrectomy specimen in March 2023. The whole gastrectomy specimen was paraffin-embedded, and serial sections revealed no persistence of tumor cells. In the image, you can note moderate atrophy of the gastric crypts with some inflammatory infiltration and slight fibrosis of the submucosa and the media (hematoxylin-eosin, ×100).

In March 2023, a subtotal gastrectomy + D2 lymphadenectomy + omentectomy + segmental resection of the splenic flexure of the colon was performed. The pathological study of the surgical specimen showed the absence of tumor cells in both the primary location and lymph nodes ypT0N0 (28/0) (Figure 2).

Maintenance of nivolumab for one year was completed in April 2024, when the last CT scan showed no signs of recurrence, and CEA, CA 19.9, and CA 12.5 tumor marker levels were within the normal ranges.

## Discussion

There are no validated predictive biomarkers for patients with gastroesophageal cancer who receive perioperative chemotherapy, and current patient selection is based purely on preoperative radiologic staging. Nevertheless, post-hoc subgroup analysis from the MAGIC trial [8] suggested that patients with dMMR/MSI-H may not benefit from perioperative chemotherapy. Furthermore, individual patient data meta-analysis focused on the value of MSI-H as a biomarker in gastric cancer, not using taxanes, and did not show a benefit of perioperative chemotherapy for patients with MSI-H gastric cancer [9]. On the contrary, the PROSECCO study based on real-world data suggests that the perioperative FLOT regimen is effective in both MSI-H and microsatellite stability (MSS) localized gastric adenocarcinoma being progression-free and overall survival longer in the MSI-H subgroup [10].

The role of anti-programmed death-1 (PD-1) inhibitors in combination with chemotherapy in HER-2-negative advanced gastric cancer has recently been established in several phase III trials, showing a relevant improvement in progression-free and/or overall survival [7,11,12]. Nowadays, its role in the neoadjuvant setting in localized gastric cancer is under research, and no drugs have been approved so far. The results of several phase II and III trials, including immunotherapy with or without chemotherapy, suggest a promising future role of immunotherapy in this setting, especially in MSI-H localized gastric adenocarcinomas. The French phase II GERCOR/NEONIPIGA study [13] using neoadjuvant nivolumab plus ipilimumab and adjuvant nivolumab and the German and Swedish phase IIb DANTE study [14] comparing perioperative FLOT to the same scheme plus atezolizumab followed by adjuvant atezolizumab, both showed higher rate of pathologic complete response (pCR) in the experimental arm. In the double-blind KEYNOTE-585 phase III study, the addition of pembrolizumab to chemotherapy significantly increased the pCR rate, mainly in MSI-H tumors (37.1%). The event-free survival increase was only statistically significant when pembrolizumab was associated with FLOT chemotherapy compared with chemotherapy alone but not with other schedules of perioperative chemotherapy [15]. Preliminary results of the MATTERHORN study showed that the addition of durvalumab to FLOT chemotherapy significantly increased the pathological response rate from 7% to 19% by central review assessment [16].

Our patient’s tumor was stable on CT after neoadjuvant FLOT and unresectable at surgery. Based on the results of the CheckMate 649 study [7], nivolumab was initiated and associated with FOLFOX chemotherapy, and the radiological response observed prompted our multidisciplinary team to re-evaluate the surgical approach. Not only complete resection was feasible, but a pCR was achieved. A major question in our case report would be whether chemotherapy played a role in obtaining such a great response. Most of the perioperative studies included chemotherapy together with immunotherapy in their designs, but the GERCOR/NEOPINGA study [13] in the perioperative setting and the subgroup analysis of the KEYNOTE 062 for MSI-H population in the advanced disease [17] suggest that immunotherapy alone may be an alternative for these patients. A similar case report to ours was reported by Hidaka et al. in 2022 [18]. A clinically unresectable stage III MSI-H gastric adenocarcinoma received pembrolizumab monotherapy after progression on oxaliplatin-based chemotherapy and intolerance to one cycle of paclitaxel plus ramucirumab. A distal gastrectomy plus D-2 lymphadenectomy was performed after six cycles of pembrolizumab, and in the pathological specimen, no vial tumor cells remained [18].

Adjuvant immunotherapy after a complete pathological response to preoperative immunotherapy in gastric cancer is not a standard of care due to the absence of data from phase III trials. In our case, the decision to offer adjuvant nivolumab was based on favorable results of adjuvant immunotherapy in MSI-H melanoma and colon cancer [19,20] and after discussion with the patient.

## Conclusions

We report the achievement of a pathological complete response after chemoimmunotherapy with FOLFOX-nivolumab in a patient with MSI-H locally advanced gastric cancer that had had previously poor response to chemotherapy alone.

It is likely that chemoimmunotherapy or immunotherapy alone may be alternative neoadjuvant treatment choices for MSI-H locally advanced gastric cancer patients. 
